# Photoacoustic Imaging Probes for Theranostic Applications

**DOI:** 10.3390/bios12110947

**Published:** 2022-11-01

**Authors:** Cailing He, Jiayuan Zhu, Huayue Zhang, Ruirui Qiao, Run Zhang

**Affiliations:** Australian Institute for Bioengineering and Nanotechnology, The University of Queensland, Brisbane 4072, Australia

**Keywords:** photoacoustic imaging, contrast agent, nanomaterials, small organic dyes, theranostics

## Abstract

Photoacoustic imaging (PAI), an emerging biomedical imaging technology, capitalizes on a wide range of endogenous chromophores and exogenous contrast agents to offer detailed information related to the functional and molecular content of diseased biological tissues. Compared with traditional imaging technologies, PAI offers outstanding advantages, such as a higher spatial resolution, deeper penetrability in biological tissues, and improved imaging contrast. Based on nanomaterials and small molecular organic dyes, a huge number of contrast agents have recently been developed as PAI probes for disease diagnosis and treatment. Herein, we report the recent advances in the development of nanomaterials and organic dye-based PAI probes. The current challenges in the field and future research directions for the designing and fabrication of PAI probes are proposed.

## 1. Introduction

The advancement of non-invasive imaging technologies, including ultrasound imaging (UI), computed tomography (CT), positron emission tomography (PET), magnetic resonance imaging (MRI), Raman imaging (RI) and fluorescence imaging (FI), is contributing significantly in biomedical research and clinical investigation [1]. Among them, UI can realize real-time imaging, CT can provide three-dimensional imaging and MRI shows high spatial resolution [2]. Although such modalities have many attractive merits and hold tremendous potential for clinical imaging and diagnosing diseases, several disadvantages cannot be ignored, such as the limitation in quantitative analysis, low spatial resolution, and shallow penetration. As such, researchers are devoted to developing new imaging technologies and photoacoustic imaging (PAI) is a promising candidate [3,4,5]. As of the negligible scattering of acoustic waves, PAI can retain a high spatial resolution and deep penetrability in biological tissues. In contrast to ionizing radiation-mediated PET and CT, PAI is much safer with the acoustic signals whereas providing higher imaging contrast between diseased and healthy tissues [3,4].

**Figure 1:**
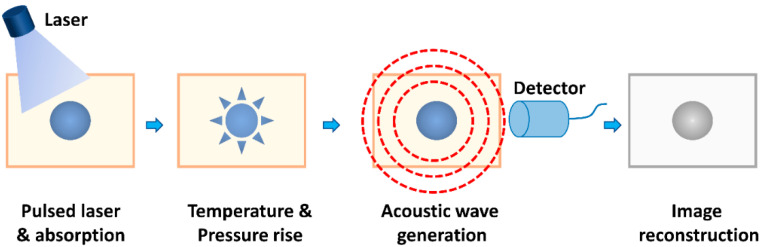
Schematic illustration of the principle of photoacoustic signal generation, detection, and image formation.

The first photoacoustic (PA) effect was described by Alexander Graham Bell in 1880 [6]. In following studies, Lloyd Barton Kreuzer pushed the limits of high peak power and high spectral purity of pulsed lasers in 1971, which facilitated the imaging application of PA [7]. Subsequently, PAI attracted increasing attention in a spectrum of biomedical fields, especially in disease theranostic applications [8,9]. PAI, an emerging biomedical imaging technology, capitalizes on a wide range of endogenous chromophores and exogenous PA contrast agents to offer functional and molecular information related to diseased tissues [4]. As shown in Figure 1 of the general principle of PAI, PA effect is a process of generating acoustic waves which result from absorbed optical energy under a nano-second pulsed laser [2]. PAI leverages the PA effect to acquire detailed real-time information of the cellular and molecular signatures of biological tissues. Specifically, after absorbing laser energy, endogenous and exogenous contrast agents attributed to the transient expansion of biological tissues due to the temperature increase and such temperature changes lead to the generation of ultrasound waves. Afterwards, the ultrasound transducer was used to measure as well as process the acoustic waves and form an image [10].

It is unavoidable that with the increasing depth of biological tissues, the light intensity and PA signal-to-noise ratio show a dramatic attenuation because of the light scattering effect. One of the efficient ways to overcome this problem is to use high performance exogenous chromophores to enhance the imaging contrast [11,12]. As such, the development of exogenous contrast agents has attracted enormous attention in PAI research in recent years [12,13,14]. Generally, a commendable contrast agent must have several features, including low quantum yield, high extinction coefficient, near-infrared (NIR) window peak absorption, great photostability, good target affinity, specificity, and biocompatibility [15,16]. Endogenous chromophores, including oxyhemoglobin, lipids and collagen, provide functional and anatomical information and exogenous contrast agents, from small molecular organic dyes to metallic nanomaterials, aid in cellular and molecular events in PAI [11,12,17]. To date, a huge number of exogenous chromophores have been designed for PAI [18,19]. Herein, we review popular exogenous contrast agents as the PAI probes ranging from nanomaterials to small molecular organic dyes, followed by the applications of these probes for disease diagnosis and treatment. Current challenges and future research perspectives of the development of PAI theranostic probes are then discussed.

## 2. Nanomaterials-Based PAI Probe

Functional nanomaterials with NIR light absorption and thermal expansion properties have been widely used as the probes for PAI in biological and medical research. To date, several nanomaterials, including gold nanomaterials, metal (e.g., palladium and copper) based nanomaterials, carbon and its combined metallic nanomaterials, and semiconducting polymer nanoparticles, have been developed as the PAI exogenous contrast agents for biomedical research and (pre)clinical diagnosis and treatment, which are summarized in this section.

### 2.1. Gold Nanomaterials

**Figure 2:**
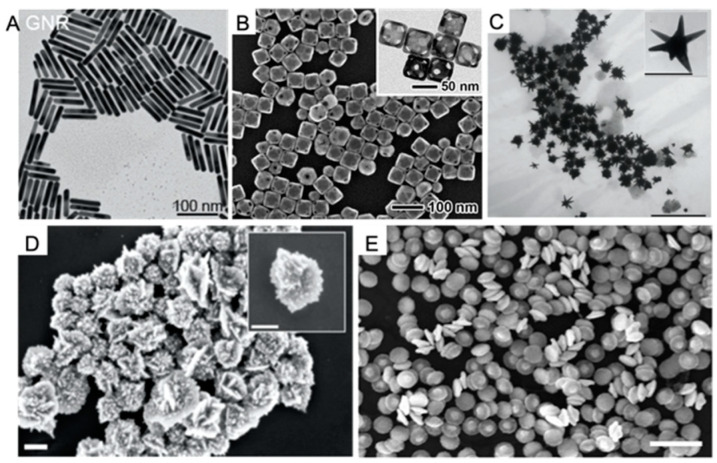
TEM and SEM images of gold nanomaterials for PAI. (**A**) TEM image of GNRs. Reproduced with permission from Ref. [20] Copyright 2021, American Chemical Society. (**B**) SEM images of GNCs. The inset shows the corresponding TEM images of the same sample. Reproduced with permission from Ref. [21] Copyright 2011, Wiley. (**C**) TEM image of dual plasmonic gold nanostars (DPGNSts) (scale bar: 500 nm) and high-magnification image of single DPGNSts (scale bar: 100 nm). Reproduced with permission from Ref. [22] Copyright 2016, Wiley. (**D**) SEM images of GNFs (scale bars: 100 nm). Reproduced with permission from Ref. [23] Copyright 2021, American Chemical Society. (**E**) SEM image of GNDs (scale bar: 500 nm). Reproduced with permission from Ref. [24] Copyright 2017, American Chemical Society.

Gold nanomaterials, including gold nanoparticles (GNPs), gold nanorods (GNRs), gold nanocages (GNCs), gold nanostars (GNSts), gold nanoflowers (GNFs) and gold nanodisks (GNDs) (Figure 2), have been the key contrast agents for PAI. Different from the small molecules based PAI probes, the surface plasmon resonance (SPR) effect of gold nanomaterials offers them strong light absorption and thus higher PA signals in imaging. The size and shape of gold nanomaterials that are related to the resonant frequency are easily tuned to be in the near-infrared window between 650 and 1350 nm, where the attenuation in blood and tissues is minimum. Moreover, the high surface area of nanomaterials sheds light on the versatile chemical modifications, facilitating the biological applications. In this section, advances in gold nanomaterials, such as in PAI probes, including GNRs, GNCs, GNSts, GNFs, and GNDs, are summarized.

#### 2.1.1. Gold Nanorods (GNRs)

**Figure 3:**
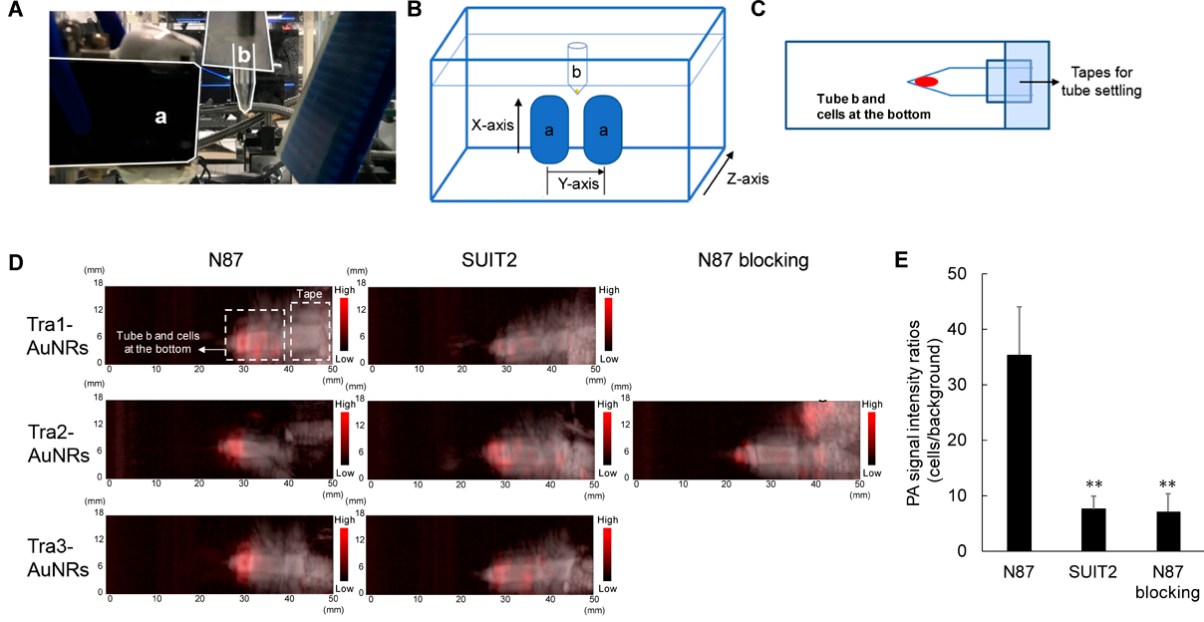
Photoacoustic equipment and PA signals after Tra-AuNRs incubation with HER2 positive and negative cells. (a) Light illuminator and ultrasound-wave receiver, (b) A sample tube with cells at its bottom was hung over the water tank. (**A**) Instrument of the PA imaging; (**B**,**C**) Corresponding diagrams of the instrument; (**D**) In vitro PA imaging study of Tra1-, Tra2-, and Tra3-AuNRs using both N87 and SUIT2 cells after 6 h of incubation; (**E**) PA signal intensity ratios of Tra2-AuNRs-added cell samples, ** *p* < 0.01. Reproduced with permission from Ref. [27] Copyright 2020, J-STAGE.

GNRs possess two plasmon modes, longitudinal and transverse localized surface plasmon resonance (LSPR). The precise tuning of GNRs aspect ratios (ARs) enables the plasmon band in the range from visible to NIR regions [25]. In PAI and other biomedical applications, GNRs can be easily modified with numerous different layers, facilitating the imaging performance [26]. For example, Ding and co-workers conjugated trastuzumab, a drug of anti-human epidermal growth factor receptor 2 (HER2), with radioisotope (^111^In) labelled GNRs (^111^In-Tra-GNRs) [27]. The PAI results indicate that the Tra-GNRs stained human gastric cancer (N87) cells presented higher PA signals than human pancreatic cancer (SUIT2) cells. As a result of the ^111^In labelling, the images of in vivo single-photon emission computed tomography (SPECT) demonstrated that the amount of N87 cells was largely increased in comparison with that of SUIT2 cells (Figure 3). These images suggested the great potential of Tra-GNRs in SPECT and PAI for tumor detection and treatment.

In 2021, Yim and co-workers developed a melanin-GNRs with an easy-tuned polydopamine (PDA) protective coating [20]. Such coating endowed GNRs with great light absorption and thermal stability, resulting in three times higher PA signal than normal GNRs. In vivo studies revealed that PDA-coated melanin-GNRs showed low toxicity and favorable colloidal stability, promoting their application in PAI in biomedical samples. Through detecting upregulated microRNA-21 expression in tumors, Yan et al. reported a FIRE-PEI-GNRs (FIRE: fuel improved microRNA explorer, PEI: polyethyleneimine) system for discriminating tumor tissues from normal cells [28]. This system with PAI capability showed high sensitivity for effective detecting microRNA-21 at tumor cells, and the increased temperature from 30 to 52 °C of the FIRE-PEI-GNR probe under 808 nm NIR laser resulting in excellent photothermal effect in vitro and in vivo with 23.7% photothermal conversion efficiency.

#### 2.1.2. Gold Nanocages (GNCs)

GNCs with single-crystal structure, hollow interiors as well as ultra-thin and multihole wall [21], have been ideal candidates for PAI contrast agents. To a better understanding of the distribution and cleavage sites of protease in tumor tissue, in 2018, Liu et al. reported the development of a multispectral PAI activatable probe, GNC-peptide-fluorescent Alexa Fluor Dye680 (Dye680) (GPD) [29]. After activating by protease MMP-2, the peptide linker was cleaved to release the GNCs (PA770) and Dye680 chromophore (PA680). Because of the different retention speeds of these two compounds, the obvious change in PA signal was detected that was then used to confirm the distribution of cleavage sites and develop protease imaging in vivo. In 2019, Xu et al. reported the preparation of HA-modified GNCs (GNCs-HA) as a theranostic-related nanoplatform for a targeted tumor PAI and treatment [30]. In this GNCs-HA nanosystem, HA served as the breast cancer targeting unit [31] and GNCs served as a contrast agent of PAI and photothermal therapy (PTT). In a 4T1 tumor model, the tumor tissues showed remarkable higher PA signals than other tissues after 24 h GNCs-HA injection. Moreover, combination treatment of 4T1 tumor by X-ray mediated radiotherapy, photothermal and photodynamic therapies (PTT/PDT) was then demonstrated, suggesting GNCs-HA is promising in PAI-guided tumor diagnosis and combination treatment.

#### 2.1.3. Gold Nanostars (GNSs)

**Figure 4:**
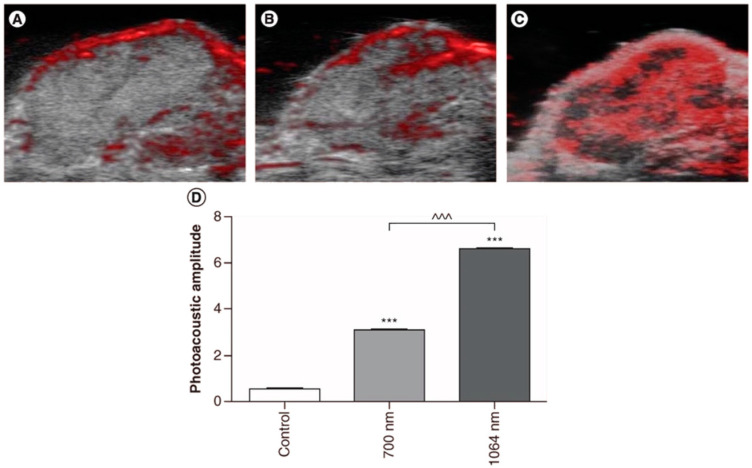
(**A**) PA images of untreated tumor; (**B**,**C**) Tumors administered with DPGNS and PA scans acquired with 700 and 1064 nm. The circles indicate the region of interest in tumors; (**D**) Plot showing the PA amplitudes of the images in (**A**–**C**), *** *p* < 0.001 versus control; ^^^ *p* < 0.001 versus 1064 nm wavelength. Reproduced with permission from Ref. [35] Copyright 2017, Future Medicine.

As of the desirable photothermal conversion capabilities and low-cost synthesis process, GNSs have recently been increasingly used in the fields of PAI and nanomedicine [32]. In 2015 Liang and co-workers reported a CD44v6 monoclonal antibody loaded PEG-GNSs (GNS-PEG-CD44v6) for cancer PAI and treatment [33]. This nanoplatform showed high stability, good biocompatibility and photothermal ablation capacity. A 4.7-fold increase in PA signal was observed in the tumor tissues after 4 h injection of the GNS-PEG-CD44v6, suggesting its high tumor tissue targeting capability. In comparison with inducting tumor stem cell differentiation, this nano-platform is more effective to hamper the proliferation and invasion of tumor, suggesting promising theranostic application. Using gallic acid (GA) as the reductant, Zhang et al. reported the size and shape controllable preparation of GNSs for PAI and imaging-guided PTT in 2016 [34]. Subsequently, Raghavan and co-workers reported DPGNSts with two different LSPR bands (700 and 1100 nm) and silica coating for enhanced PAI (Figure 4) [35]. In 2020, Li et al. demonstrated a chlorin e6 (Ce6) and polydopamine (PDA) coupled GNSs (Ce6/PDA-GNSs) to achieve PAI-guided PTT and PDT for the inhibition of breast cancer growth [36]. In contrast with the bare GNSs, the PDA coating and Ce6 loading improved the nanoplatform’s PAI capability and PTT performance, and offered the nanosystem PDT capacity. The experimental results clearly indicate that Ce6/PDA-GNSs can inhibit the growth of 4T1 tumor cells and restrain their metastasis. To improve the photostability of GNSs, Chen et al. coated the GNSs with silica at different shell thicknesses [37]. The result showed that a 25 nm-thick silica shell coated GNSs maintain their shape after laser irradiation. The improved PA stability allowed the GNSs with silica shell for long-term PAI in vivo.

#### 2.1.4. Other Gold Nanoparticles

Other gold nanomaterials, such as GNFs, GNDs and GNPs have also been developed as probes for PAI. In 2021, Santos et al. were committed to tuning the optical property of GNFs from 590 to 960 nm for PAI and hyperthermia [23]. The prepared GNFs showed high stability in cell culture media, and low toxicity to mouse fibroblast (FC3H) cells while exhibited high efficiency in tumor PTT. The PAI results reveal that the GNFs could be used for imaging even at low concentrations. Through a physical method to vacuum deposit Au onto a polymer nano-layer, the two-dimensional (2D) GNDs with stacked structures and different sizes were prepared by Wi et al. in 2017 for PAI and optical coherence imaging [24]. These GNDs have the intrinsic optical advantages of a wide range of resonant wavelengths, the tunable ratio of light absorption-to-scattering, and responsiveness to random incident light. The application of these GNDs for improved PAI was then successfully demonstrated. Feng et al. developed a Cu^2+^-activated nano-gapped GNPs (NGNPs) PA probe to detect Cu^2+^ in liver to early diagnose Wilson’s disease (WD) [38]. After receiving an injection of 100 mL PA nanoprobe, the PA_1250 nm_ signal intensity reached the peak in WD mice at 14 h. Based on the linear relationship between PA_1250 nm_ and Cu^2+^ concentration, the liver Cu^2+^ level in WD mice was calculated to be 3.052 ± 0.045 mm, which is confirmed by ICP- MS test results (3.054 ± 0.027 mm), suggesting the good Cu^2+^ quantitative measurement ability of this PA nanoprobe in vivo.

### 2.2. Other Metallic Nanomaterials

In addition to GNPs, other metallic nanomaterials, such as palladium (Pd) nanosheets, copper sulfide nanoparticles, have also been developed as the contrast agents for PAI. In 2011, Zheng’s group reported the preparation of 2D Pd nanosheets with plasmonic and catalytic properties. The nanosheet with increasing edge length from 21 to 27, 41 and 51 nm showed redshifted SPR peaks from 826 to 992, 1045 and 1068 nm, respectively [39]. In a following study in 2016, the same research group discussed the safety of the Pd nanosheets and their application in PTT and PAI [40]. A series of Pd nanosheets with different diameters from 5 to 80 nm were prepared and corresponding PAI capabilities of tumor were investigated. The results show that all Pd nanosheets can be used for PAI, while the contrast effects of smaller-sized (5, 13, and 30 nm) Pd nanosheets were superior to that of 80 nm Pd nanosheets because of the reduced tumor accumulation of the 80 nm Pd nanosheets.

In 2015, Ding and co-workers prepared PEGylation conjugated copper sulfide NPs (PEG-CuS-NPs) with small particle size (3–7 nm) for PAI of tumor [41]. PEG-CuS-NPs showed low cell toxicity and high colloidal stability. The PAI images revealed that the PEG-CuS-NPs smaller than 5 nm exhibited higher tumor imaging capability, especially at the tumor boundary area, suggesting the potential of PEG-CuS-NPs as an excellent PA contrast agent for in vivo PAI-guided tumor therapy.

**Figure 5:**
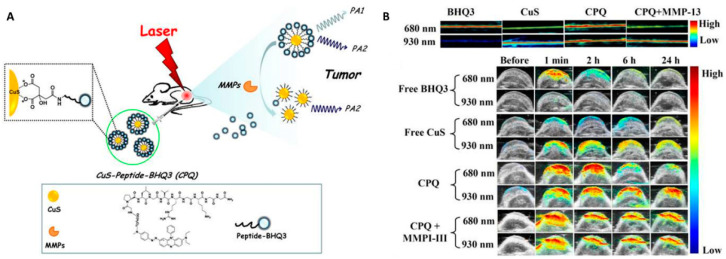
The development of CuS-based activatable PAI probe for MMP detection and visualization in tumor. (**A**) The principle of the MMP-responsive PAI probe. (**B**) PAI ratiometric detection of MMP in solution (**top**) and tumor tissues (**bottom**). Reproduced with permission from Ref. [43] Copyright 2014, Ivyspring International Publisher.

Matrix metalloproteinases (MMPs) contribute to degrading proteins in the extracellular matrix (ECM) and thus have a positive effect on the treatment of a variety of diseases [42]. In 2013, a smart activatable PAI probe was developed by Yang et al. for the detection and visualization of MMP activity in vivo [43]. As shown in Figure 5, the probe was designed by attaching a black hole quencher 3 (BHQ3) linked peptide on the surface of CuS nanoparticle. The probe showed two absorptions at 680 and 930 nm (PA680 and PA930, respectively). In the presence of MMPs, the cleavage of the peptide led to the release of CuS nanoparticles and BHQ3 molecules. The PA680 was quickly diminished while that of PA930 was largely retained, allowing ratiometric PAI of tumor tissues.

### 2.3. Carbon-Based Nanoparticles

**Figure 6:**
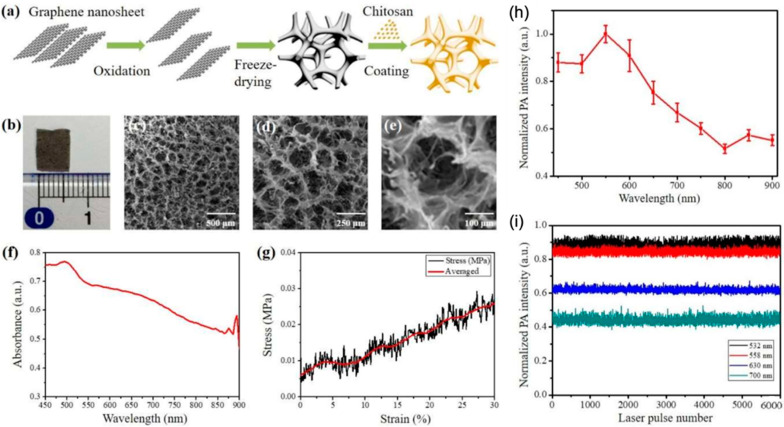
Preparation route, characterization and PA performance of the Chi-GO hemostatic sponge. (**a**) Preparation route of hemostatic sponge; (**b**) Photograph of the cross-section of the sponge; (**c**–**e**) SEM image of the porous structure of the sponge at indicated magnification scale; (**f**) Absorbance spectrum; (**g**) The stress–strain curve in the mechanical compression test; (**h**) Measured PA spectra, (**i**) Photostability test at indicated wavelength. Reproduced with permission from Ref. [46] Copyright 2021, MDPI.

Carbon-based nanomaterials, particularly graphene, have also been explored as the contrast agents for PAI. Graphene is a 2D semiconductor nanomaterial that displays ultrafast optical and electrical properties for highly sensitive and speed surface wave detection [44]. Their optical properties enabled graphene to be used as a potential candidate for PA probes [45]. In 2018, Yang and co-workers developed a highly sensitive broadband PA probe for in vivo label-free PAI [44]. Based on this nanostructure, in vivo microvasculature three-dimensional (3D) images of a mouse ear were conducted. In 2019, through conjugating folic acid (FA) on chitosan-functionalized graphene oxide nanomaterial, a PAI nanomaterial was developed by Lin and colleagues for the imaging of tumor in a mice model [46]. This chitosan–graphene oxide (Chi-GO)-based hemostatic sponge was tested for wound healing, and the results show that this material has fantastic absorption of blood plasma and induces interfacial reaction with platelets and erythrocytes too (Figure 6). Wang’s group reported a reduced graphene oxide (rGO) with GNPs coating for PAI [47]. The GNPs on the surface promoted the contrast of rGO for imaging of tumor tissues and displayed an excellent PTT effect after a 1061 nm laser irradiation in SKOV-3 tumor-bearing mice. In 2020, a rGO@GNS with lipid surface nanostructure was also developed for PAI-guided tumor therapy in pancreatic cancer, showing 66.3% photothermal conversion efficiency under 808 nm irradiation [48]. The folic acid on the surface of the material also contributed this material to be a gene carrier, as well as allowed the targeting of tumor cells for PAI and PTT/gene synergistic therapy [46,48]. The in vivo results from Jia et al. show that PTT/gene group had 98.5% tumor growth inhibition rate, much higher compared to PTT (76.1%) or gene therapy (55.2%) only treated group depending on 808 nm laser (1.2 W cm^−2^) or K-Ras short hairpin RNA expression plasmid (Krasl) [48].

Based on their strong light absorption in NIR and deep tissue penetration, carbon nanotube (CNTs) have been investigated for various biomedical applications, such as PA and in vivo photothermal imaging [49]. In 2014, Wang et al. covalently conjugated carboxyl groups of multiwalled carbon nanotubes (MWNTs) with silica-modified gold nanorods (sGNRs) to develop RGD-sGNRs-MWNTs for targeted tumor PAI and treatment, in which the RGD peptides contributed to the tumor cells’ targeting and sGNRs-MWNTs served as the PAI contrast agent [50]. The images showed that the PA signals at 800 nm were enhanced after integrating of sGNR with MWNTs, suggesting the great potential of RGD-sGNRs-MWNTs for tumor-targeted imaging. This carbon-based material, as a multimodal PA and photothermal contrast agent, has been demonstrated to be potential in the tumor theranostic application.

### 2.4. Semiconducting Polymer Nanoparticles

**Figure 7:**
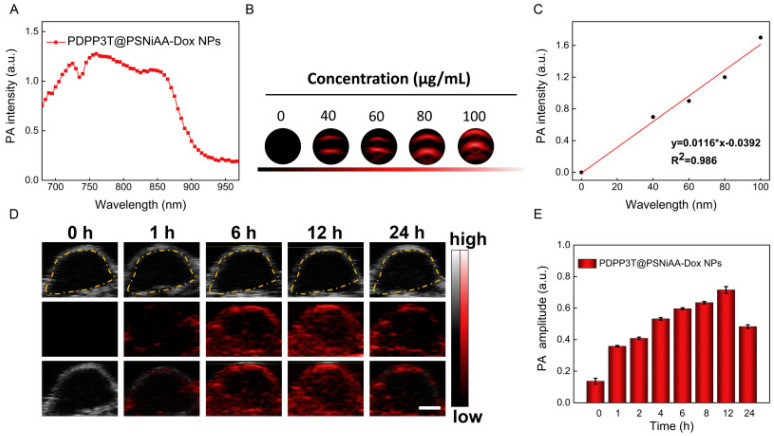
PA properties of PDPP3T@PSNiAA−Dox NPs in vitro and vivo. (**A**) PA spectrum of PDPP3T@PSNiAA−Dox NPs; (**B**) The PA images of PDPP3T@PSNiAA−Dox NPs at various concentrations in low−PA−density tubes; (**C**) The relationship of PA intensity and concentration of PDPP3T@PSNiAA−Dox NPs; (**D**) In vivo PA images and intensity of PDPP3T@PSNiAA−Dox NPs; (**E**) In vivo PA signal of PDPP3T@PSNiAA−Dox NPs in tumor area as a function of postinjection time. The scale bars of all images are 2 mm. Reproduced with permission from Ref. [50] Copyright 2019, Elsevier.

Semiconducting polymer nanoparticles (SPNs) are another new photonic biomaterial with great potential for theranostic applications. SPNs are designed to improve the sensitivity and resolution of PAI because of the tunable optical properties, high absorption coefficients and high photostability of this material [51]. In 2019, through reversible addition fragmentation chain transfer (RAFT) polymerization of polystyrene-b-poly (N-isopropylacrylamide-co-acrylic acid) (PSNiAA), Xu’s team prepared a functional nanoparticles (PDPP3T@PSNiAA) with photothermal sensitivity and PA response [52]. Anti-cancer drug Doxorubicin (Dox) was also loaded onto these NPs by electrostatic interaction and formed PDPP3T@PSNiAA-Dox NPs. The data showed that all PDPP3T@PSNiAA-Dox NPs at different concentrations had a time-dependent increasing laser irradiation response. The temperature of the NPs could still increase to the same level even up to the fourth laser-cooling circle, indicating the photothermal stability of PDPP3T@PSNiAA-Dox NPs. Additionally, experiments for the PA properties of these NPs conducted in vitro showed that the maximum intensity of PDPP3T@PSNiAA NPs was at 785 nm, and the higher concentration of NPs contributed to higher PA signals. The in vivo experiments indicated that these PDPP3T@PSNiAA-Dox NPs could accumulate in tumor region over time (Figure 7). A burst intracellular Dox release occurred with NIR irradiation at acidic condition (80%), while it was below 40% at neutral condition, which could minimize the release of the drug in normal tissues. In vivo studies on HeLa tumor-bearing mice showed that tumors of PDPP3T@PSNiAA-Dox NPs with laser treatment were effectively eliminated without reoccurrence, while groups treated with free Dox (with and without laser) and NPs without laser showed none or minor tumor elimination. The pH/NIR light-triggered release of Dox made PDPP3T@PSNiAA-Dox NPs a promising probe for PAI-guided PTT/chemo-combined theranostic application.

In 2020, Zheng et al. reported SPN coated with the red blood cell membrane (SPN@RBCM) could be used for PAI and PTT [53]. Based on the particularly small size (<5 nm), these nanoparticles could reach deep tumor tissues and be cleared from human body rapidly with no noticeable toxicity [53]. In another work, Bindra and his colleagues developed a self-assembled nanoagent (SP-CS) containing semiconducting polymer (SP) and encapsulated copper sulfide CuS (CS) nanoparticles, which could lead to incredible PA signals and reactive oxygen species (ROS) generation, and displayed the ability for PTT by 808 nm laser activation [54].

## 3. Small Molecular Organic Dye-Based PA Probes

The development of small molecules-based probes for imaging, e.g., FI [55,56,57,58], MRI [59], PAI [12,19], has attracted enormous interests in recent years due to their excellent excretion ability from body, easily conjugating with targeting groups, and stability in the detection and imaging in vitro and in vivo [18]. A range of PA probes have also been approved by FDA, such as indocyanine green (ICG), IR-dye fluorophores, Boron-dipyrromethene (aza)BODIPYs, cyanine dye as well as porphyrins, and their wide range of potential applications in biomedical fields have been investigated in recent years. On the basis of the organic dyes, probes for fluorescence analyses of various biomolecules, such as ONOO^-^ [60], hydrogen sulfide (H_2_S) [61,62], Cysteine (Cys) [55,63,64], nitric oxide (NO) [65,66], ROS [67,68] and hypoxia [69,70], while the number of probes for PA detection and images was limited [71,72]. The toxicity and pharmacology of gold-based nanoprobes, and azo-bridged dyes cause major constraints to their clinical translation. Porphyrins, cyanine dyes (e.g., ICG), polymethine-based cyanine dyes and cyanine-containing quencher dyes are low toxicity and high NIR absorption, could be translated for clinical applications [73]. In light of the change in the PA signal, small molecular organic dye-based PA probes mainly include (i) “on-off” small molecular organic dye-based PA probes; (ii) “off-on” small molecular organic dye-based PA probes; (iii) “always on” small molecular organic dye-based PA probe; and (iv) small molecular organic dye-based ratiometric PA probes. “On-off” PA probes, generally, have strong PA signals before the detection and demonstrate decreased PA signals in the presence of the targeted analytes, while “off-on” PA probes exhibited the opposite process of “on-off”. The one of “always on” PA probes shows stable PA signals in entire detection and imaging processes, while the ratiometric PA probes exhibit different changes of PA signal at two or more wavelengths. Compared with ‘‘always on’’ PA probes, “on-off” and “off-on” probes are able to acquire a higher signal-to-background (SBR) and lower limit of detection (LOD), contributing to the specific and sensitive diagnosis of diseases [74]. The small molecules-based PA probes, including both molecular probes and the small molecules as the core PA signaling unit are outlined in this section.

### 3.1. “On-Off” Small Molecular Organic Dye-Based PA Probes

**Figure 8:**
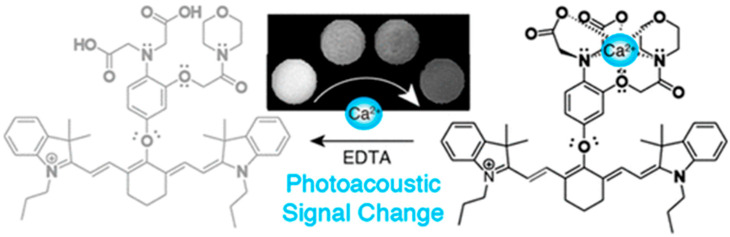
Schematic Representation of NIR Ca^2+^ Sensing PAI Probe, L, In Its Unbound and Ca^2+^-Bound Form. Reproduced with permission from Ref. [77] Copyright 2016, American Chemical Society.

For “on-off” PA probes, the interaction between probes and biomarkers in target tissues reduces probes’ light absorption and leads to the decrease in PA signals [75]. Divalent cations play crucial roles in a wide range of biological activities in body, such as DNA and myelin synthesis, redox processes, and oxygen transportation. Calcium (Ca^2+^) is available for central synaptic transmission, muscle contraction, apoptosis, and inhibiting the proliferation of cancer cells [76]. To determine Ca^2+^, Mishra et al. reported an “on-off” NIR-PA probe L which was composed of heptamethine cyanine dye (IR-780) and a Ca^2+^-responsive chelator (Figure 8) [77]. When reacting with Ca^2+^, the absorbance at 765 nm of this NIR-PA probe L demonstrated a significant decrease, leading to the obvious change in PA signals from “on” to “off”.

**Figure 9:**
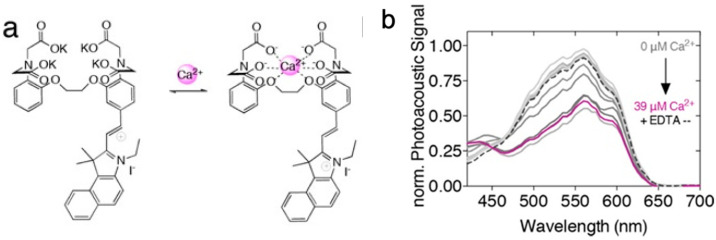
(**a**) Chemical structure of CaSPA in its unbound (left) and Ca^2+^-bound (right) forms; (**b**) PA analysis of CaSPa (25 μm) dissolved in MOPS (2 mm) buffer containing increasing concentrations of free Ca^2+^ (0−39 μm). Reproduced from permission from Ref. [78] Copyright 2018, American Chemical Society.

In another work, Roberts and co-workers coupled Ca^2+^ chelator BAPTA with fura-2 chromophore as the probe (CaSPA) for PA detection and imaging of Ca^2+^ [78]. With the increase in Ca^2+^ level, a blue shift of the absorption spectra was observed, resulting in the decrease in PA signals (Figure 9). Moreover, the experiments implied that CaSPA was highly applicable for the live imaging of Ca^2+^ in cells and the zebrafish brain by the combination of PAI and fluorescence.

**Figure 10:**
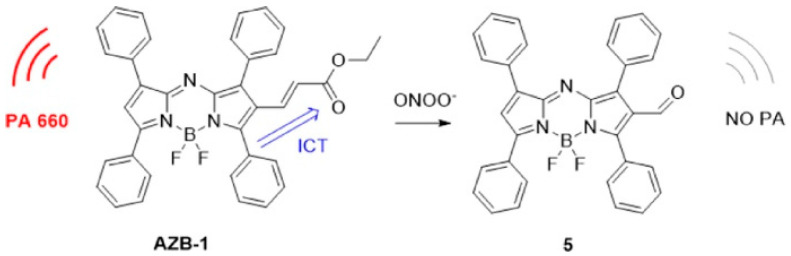
Design concept and structures of AZB-1 and 5. Reproduced with permission from Ref. [79] Copyright 2021, Elsevier.

In 2021, Ma and co-workers introduced an unsaturated ketone group at 2-position of aza-BODIPY to synthesize the AZB-1 for ONOO^-^ detection and imaging in vivo (Figure 10) [79]. Such probe had strong PA signals at 660 nm, but the reaction between AZB-1 and ONOO^-^ led to the break of AZB-1 conjugated system, resulting in an “on-off” PA response to ONOO^-^. The application of this probe was then demonstrated by PAI of ONOO^-^ generation in rheumatoid arthritis (RA), in which the mice model was established by injecting λ-carrageenan [80]. The imaging results show that the PA signal at the RA limb was about 3 times lower than that of the normal limb, suggesting the potential of AZB-1 for imaging of ONOO^-^ generation in vivo.

**Figure 11:**
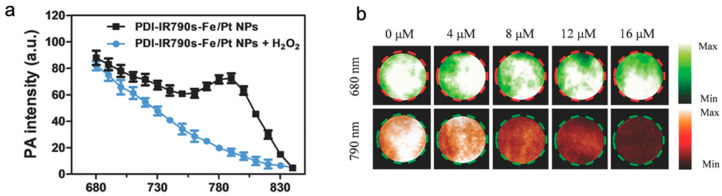
(**a**) Representative PA spectra of the PDI-IR790s-Fe/Pt NPs with or without H_2_O_2_ treatment; (**b**) PA images of the PDI-IR790s-Fe/Pt NP solution at 680 and 790 nm treated with increased concentrations of H_2_O_2_ (from right to left). Reproduced from permission from Ref. [83] Copyright 2018, Wiley.

ROS, such as hydrogen peroxide (H_2_O_2_), superoxide anion (O_2_^•−^), and hypochlorous acid (HOCl), are normal by-products of numerous cellular processes, are often associated with cancer cell homeostasis [81,82]. To determine the concentration of ROS and regulate the ROS concentration for treatment, Yang et al. developed a ROS-responsive NP platform to treat tumors and real-time monitor the therapeutic process via PAI in 2018 [83]. The NP platform (PDI-IR790-NP) possessed two key parts, including cisplatin as the antineoplastic drug to trigger the increase in ROS level and perylene-diimide (PDI) and IR790s to generate PA signals. Among them, IR790s, acting as a ROS-sensitive small organic dye, was decomposed when reacting with ROS. As a result, the decrease in PA signals at 790 nm was observed for ROS determination (Figure 11).

Rao’s group has also reported several polymer-based nanoprobes for PA detection and imaging [84,85,86,87]. Recently, a reversible photoswitching upconversion nanoprobe was developed by Rao’s group for super-sensitive PAI [88]. The photochromic diarylethenes was loaded on the surface of upconversion nanoparticles (UCNPs) for fabricating the nanoprobe. The emission of UCNPs allowed the photochromic response and the emergence of diarylethenes’ absorption facilitated the reversible “off-on-off” PA signal for imaging. With this UCNPs-mediated PAI system, the background PA signals from endogenous chromophores were minimized. In 2022, Chan et al. reported a reversible PA probe based on 3,5-di-tert-butyl-4-hydroxyphenyl-substituted boron dipyrromethene (DiOH-BDP) and can be utilized to detect ClO^−^/GSH. After injecting DiOH-BDP in acute liver injury (ALI) mouse models, a strong PA signal at 770 nm was observed because of the high level of ClO^−^. Meanwhile, the injection of lipoic acid to regenerate GSH in ALI models attributed to the recovery of DiOH-BDP and led to the decrease in PA signal at 770 nm. As a result, PA signals at 770 nm can be explored for detecting ClO^−^/GSH by using DiOH-BDP in vitro and vivo [89].

### 3.2. “Off-On” Small Molecular Organic Dye-Based PA Probes

The PA signal of “off-on” probes is absent at normal tissues and it will be activated and generate signals when interacting with targets. Companion diagnostics (CDx) is a new emerging technology to detect biomarkers that are highly related to activating the drug to prognosticate whether this specific drug is able to benefit a patient [90]. Such information enables patients to acquire individualized and targeted treatment. It has been reported that the concentration of glutathione (GSH) in cells is directly correlated to a wide range of pathologies, like lung cancer. Herein, Lucero et al. designed a PA-based CDx (PACDx) for the determination of GSH in different animal models of lung cancer [91]. PACDx contained two major parts, a hemicyanine dye (HD)-based PA signaling unit and a GSH-responsive trigger that controls the switch of PA signals. In the presence of GSH, PACDx’s PA signal intensity at 690 nm increased from 0 to 800 a.u., and such signal intensity increase was proportional to the concentration of GSH. An FDA approved prodrug PARx was then coupled with PACDx, and the produced PARx can release gemcitabine to target non-small lung tumor tissues and HD-CH_2_OH to generate PA signals after reacting with GSH in vivo. In another work, Li et al. developed an activatable hydrophilic NCTy probe for Cys detection in vivo [92]. The main group of NCTy was cyanine-like dye structure (NCTy-OH). Once reacting with Cys, NCTy without PA signals can convert into NCTy-OH and induce a strong PA signal in 695 nm at the tumor site.

**Figure 12:**
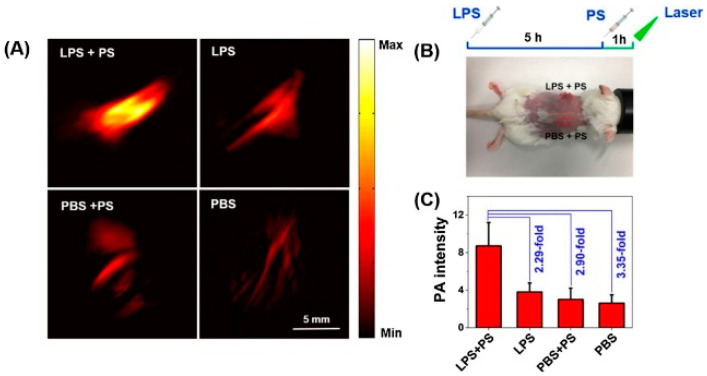
(**A**) In vivo PA images of detecting the NO generation induced by LPS in the inflamed mice model. (**B**) The locations were treated through subcutaneous injection. (**C**) PA intensity was acquired at 532 nm in triplicate for two sites, respectively. Reproduced with permission from Ref. [93] Copyright 2018, Elsevier.

A unique donor-acceptor-donor (D-A-D) structure-based “off-on” PA probe (PS-NO) for nitric oxide (NO) detection was developed by Wang et al. through coupling benzothiadiazole as the chromophore and diphenylamine as the NO recognition unit [93]. Compared with PS, PS-NO possessed the electro-attractive triazole structure implicated in obvious intramolecular charge transfer (ICT), leading to fluorescence quenching and the enhancement of PA signals. The in vivo animal experiment revealed that the PA signals were high in lipopolysaccharide (LPS) treated mice, while weak PA signals were observed for the control group, suggesting that PA signals experienced an “off-on” process in vivo because of the reaction between PS and NO (Figure 12). The above finding indicated that PS nanoprobe was highly applicable for sensitively detecting the concentration of NO via PAI and fluorescent imaging in vivo to study NO-related diseases. A similar D-A-D structure based PAI probe (QY-N) was developed by Sun et al. for NO detecting in the hepatic region [94]. QY-N showed an optical absorption peak at 670 nm. In the presence of NO, the recognition group butylamine of QY-N can react with NO (N-nitrosation reaction) to synthesize QY-NO which generated PA signals at 700–850 nm. This PA probe has been successfully applied in the detection and verification of the level of NO in liver injury in the triptolide-induced liver injury mouse model and provides a guide for the in situ drug release for the theranostic application.

Charge-transfer complex (CTC) materials contain charge-donor and charge-acceptor that possess excellent properties, including anisotropic conductivity and photoconductivity. Wang et al. fabricated a NIR II-responsive and pH-sensitive self-assembled charge-transfer (CTN) nanoprobe for PAI guided therapy [95]. Four hours after injecting with CTN probe, PA intensity at tumor site was significantly different (*p* < 0.001) from that at the normal site. This result coincided with data from the PH-sensitivity study in vitro, suggesting the good ability of CTN as a NIR II-responsive pH-sensitive nanoprobe for tumour PAI. After one year, Wang and co-workers optimised CTN nanoprobe and named stimuli-activatable nanotheranostics (SHT) for tumour detection [96]. SHT demonstrated great sensitive H_2_O_2_-activated/acid-facilitated PA signal changes in both vitro and vivo.

**Figure 13:**
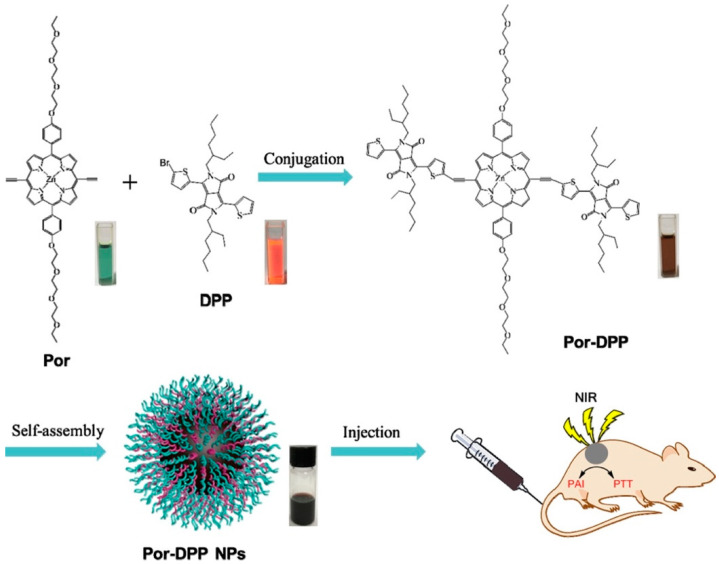
Schematic Illustration of the Preparation of Por-DPP NPs and Their Applications as Theranostic Agents for PAI-Guided PTT. Reproduced with permission from Ref. [97] Copyright 2019, American Chemical Society.

Porphyrin is a conjugated aromatic macrocycle that has high extinction coefficients and biocompatibility, but the application of porphyrin in molecular imaging is limited due to the low absorption (below 700 nm) and insufficient photostability. In 2019, Wu et al. synthesized a new porphyrin derivative with the absorption in NIR region, porphyrin-diketopyrrolopyrrole (Por-DPP) as the small molecule for the preparation of organic nanoparticles theranostic applications [97]. In aqueous solution, Por-DPP can self-assemble into nanoparticles (Por-DPP NPs) without any surfactant. After the intravenous injection of Por-DPP NPs in tumor-bearing mice, the PA intensity in tumor sites experienced a 4.3-fold increase after 8 h over the background signal. The tumor temperature raised from 31.2 to 60.8 °C after 6 min irradiation. The results suggest that such self-assembled NPs held great potential for PAI and PTT (Figure 13). Zhang and co-workers utilized the interaction between PEG and zinc porphyrin to design tri-porphyrin-based NPs with the absorption maxima up to 850 nm [98]. These NPs were used to load DOX (DOX-NPs) to simultaneously realize chemotherapy and PTT, and the information from animal experiments indicated that 12 h post-injection of DOX-NPs was the best time for PTT due to the highest PA signal intensity in tumor sites. In another work, an activatable PA probe (1-RGD) was designed by Wang and co-workers [99]. With the reaction with caspase-3 in vivo, 1-RGD self-assembled into NPs, resulting in the formation of a strong PA signal at DOX-treated tumor tissues. The specific activation of PA probes via interacting with biomarkers is beneficial to facilitate the efficacy of therapy, reduce the probe’s toxicity to normal tissues, and prompt precision medicine [74].

### 3.3. “Always on” Small Molecular Organic Dye-Based PA Probes

**Figure 14:**
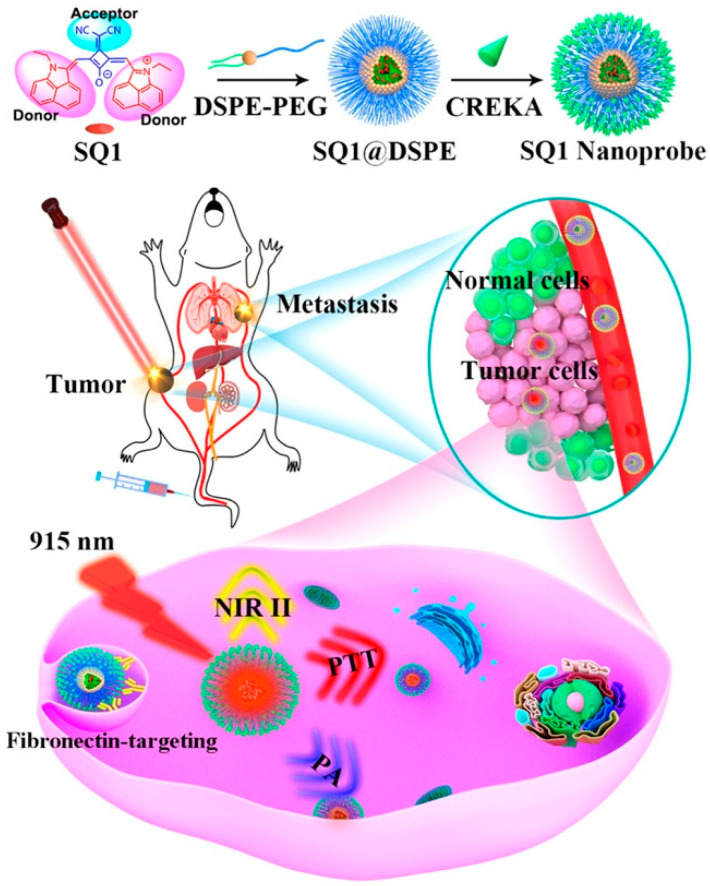
Molecular engineering and nanofunctionalization of squaraine dye SQ1 for NIR-II/PA bimodal imaging and photothermal ablation of metastatic breast cancer. Reproduced with permission from Ref. [103] Copyright 2020, American Chemical Society.

“Always on” PA probes are usually applied to the detection of biomarkers that have different concentrations in diseased and normal sites. The PA intensities of those probes keep “on” regardless of whether they succeed in interacting with the molecular of intertest or not [18]. The aza-BODIPY dye, as one of the most fascinating PAI platforms, can be modified by substituting the aryl groups on the 3 and 5 positions on phenyl rings to increase photostability and tune photophysical properties [100]. In 2020, Merkes et al. demonstrated that 1H-pyrrole conjugation to BODIPYs (PyBODIPY) can completely quench fluorescence and gain a high PA signal. The conjugation of three PEG-400 into PyBODIPY was able to adjust the water solubility, which resulted in increased blood circulation time and was beneficial to PA imaging. Such probe successfully overcame the shortcoming of BODIPY and exhibited non-phototoxic and photostable features [101]. Squaraine dyes (SQs) are a class of electron-deficient chemicals with a high molar absorption coefficient as well as excellent photoconductivity [102]. On the basis of this organic dye, Yao et al. chose to conjugate malonitrile to squaraine dye (SQ1) to improve square acid accepter and developed breast cancer and lung metastasis-double-targeting PA nanoprobe by decorating Cys-Arg-Glu-Lys-Ala (CREKA) peptide to SQ1 [103], which can indicate the overexpression of fibronectin on above-mentioned cancers (Figure 14). After injecting SQ1 nanoprobe demonstrated, robust PA signals from breast cancer (MDA-MB-231) mice models were observed, suggesting that the nanoprobes were able to effectively accumulate in tumor sites.

### 3.4. Small Molecular Organic Dye-Based Ratiometric PA Probes

The structure transformation without changing the major conjugated framework is the main feature of ratiometric PA probes. After interacting with target analytes, those probes possess a high sensitivity and show significant alteration of PA intensity [104]. In 2014, Rao’s group reported the development of a semiconducting polymer nanoprobe for PAI in living mice [105]. The NIR dyes, poly (cyclopentadithiophene-alt-benzothiadiazole) (SP1) and poly (acenaphthothienopyrazine-alt-benzodithiophene) (SP2), were loaded into the polymer nanoparticle for PAI imaging. Then, a ROS-responsive NIR dye, IR755, was co-loaded into the SP1-polymer nanoparticle for the detection and imaging of peroxynitrite (ONOO^-^) and hypochlorous acid (HOCl). The cleavage of IR755′s C=C bond led to the bleach of the PA820, while the PA700 (derived from SP1 polymer nanoparticle) was retained, allowing ratiometric PAI of ROS in mice.

**Figure 15:**
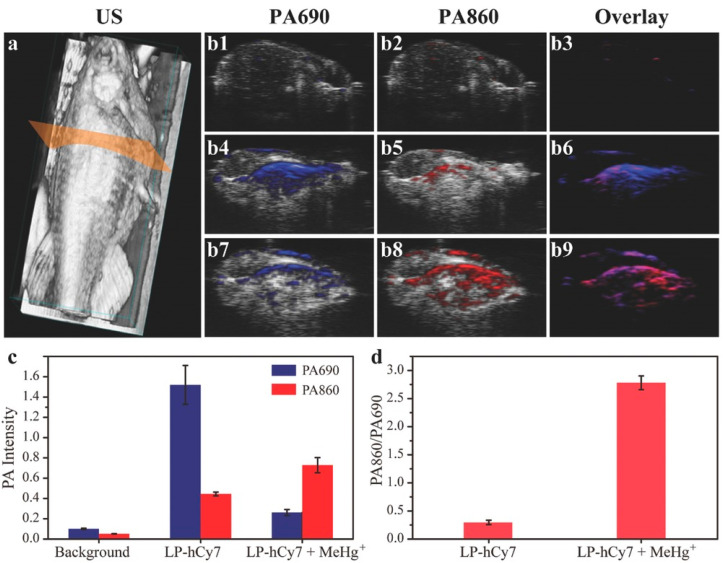
(**a**) 3D ultrasonic (US) image of zebrafish for illustration of PAI in transection of abdomen; (**b**) Merged US and PA images of untreated zebrafish (**b1**–**b3**), LP-hCy7 incubated zebrafish (**b4**–**b6**), and MeHg^+^/LP-hCy7 treated zebrafish (**b7**–**b9**) at 690 and 860 nm, respectively; (**c**) Corresponding quantified PA intensity at 690 and 860 nm for (**b**); (**d**) Ratios of PA860/PA690 obtained from (**b**). Reproduced with permission from Ref. [106] Copyright 2017, Wiley.

In 2017, Liu et al. proposed a PA nanoprobe (LP-hCy7) that had two key elements, liposome (LP) and cyanine dye (hCy7) for the detection of MeHg^+^ in vivo [106]. The main function of hCy7 was to react with MeHg^+^ to convert into hCy7′ for switching on PA signals and LP contributed to the cellular internalization process of liposoluble MeHg^+^. The PA signal intensities from the control zebrafish group (with LP-hCy7 and without MeHg^+^) were strong at 690 nm and weak at 860 nm. Nevertheless, mice injected with MeHg^+^ experienced a remarkable decrease and increase in PA signals at 690 nm and 860 nm, respectively suggesting that LP-hCy7 can be applied to detect MeHg^+^ in vivo through recording the ratiometric PA signal (PA860/PA690) (Figure 15).

In 2018, Lu et al. reported an H_2_O_2_ selective NIR PA probe which demonstrated great hydrophilicity and biocompatibility and was highly applicable for PAI in mouse models with the injection of H_2_O_2_ [107]. The probe was comprised of Aza-BODIPY for acquiring PA signals, conjugated with benzene boronic acid pinacol ester for highly responding H_2_O_2_ and hydrophilic OEG segment for enhancing water-solubility. The reaction of H_2_O_2_ with boronic acid pinacol ester led to the increase in PA825, while the decrease in PA720, resulting in ratiometric PAI of H_2_O_2_ in vitro and in A549 xenograft tumor of nude mice.

## 4. Concluding Remarks

In light of the great potential of PAI in biomedical imaging, a number of PAI probes have been developed in recent years. These probes mainly include nanomaterials and small organic dyes and corresponding small molecular dye-based nanoparticles. Among these agents, the small organic dyes exhibited excellent proprieties, such as good excretion ability and easily conjugating with target groups. Importantly, compared with nanomaterials, porphyrins and cyanine PA probes possess better application potential in clinical use due to their low toxicity. The long circulation time, capability in targeting diseases, and high PA signals of nanomaterials also allowed the PA nanoprobes to be used for imaging and the imaging-guided therapy of different diseases (e.g., PTT for cancer therapy). Furthermore, nanoparticles are also able to carry some other anticancer drugs, such as DOX and cisplatin for combination therapy, providing a unique platform for theranostic applications. This review article highlighted the recent advances of PAI probes for theranostic applications. The inorganic nanomaterials, polymer nanoparticles, and small-molecules and corresponding organic nanoparticles were discussed.

With the continuous development of PAI, more PA probes should be designed to address increasing biomedical issues by overcoming several unavoidable shortcomings. As of the endogenous contrast agents, some PA probes have high biological and low signal background ratios (S/N) during PAI in vivo [87]. Therefore, improving the sensitivity and selectivity of PA probes could be one of the key tasks in future studies. For small molecular organic dyes, these chromophores normally show insufficient photostability and PA imaging quality which cause major constraints to their application in vivo. Modification of these small molecules is thus necessary to improve the photostability and PA imaging contrast. Furthermore, the combination of PAI with other sensing and imaging techniques, such as FI, MRI, Raman can also benefit future biomedical and clinical applications for improved precision and accuracy.

Although PAI has been widely used in clinical diagnosis, the use of exogenous PAI probes remains limited. This is mainly because of the uncertain safety issues of the probes, including nanomaterials and small molecules that were summarized in this work. Therefore, the long-term safety investigations of these probes could be one of the key points for the translation of the exogenous probes in clinical diagnosis. Moreover, although the acoustic waves are highly penetrable in body, the shallow penetration of the incident light hampers the clinical application of PAI in deep tissue (over 2–3 cm deep) [108]. It has been reported that the clinically brain PAI is limited by attenuated light and ultrasound through the human skull [4]. “Delivery” of light into body to activate the PA probes could be one of the solutions. Although the UCNP has been recently used for converting the lower energy light to higher energy light for reversible PAI [88], the penetration depth of NIR light (980 nm laser) is also limited. Light “delivery” approach by other technology, such as X-ray excited luminescence nanomaterials we proposed recently could be another solution, in which the X-ray excited luminescence “nanolaser” as the local light source to activate coupled probes for PAI.

Overall, as the development of PA probes and the PAI technique are progressing gradually, we are convinced that more and more exogenous PA probes with excellent sensing and imaging capability would be developed to promoting the PAI technique in the not-too-distance future, and such technology advances would significantly benefit to future diseases early diagnosis, treatment and treatment monitoring.

## Data Availability

Not applicable.
